# Association of Computed Tomography With Treatment and Timing of Care in Adult Patients With Peritonsillar Abscess

**DOI:** 10.31486/toj.18.0168

**Published:** 2019

**Authors:** Maria C. Carratola, Gabriella Frisenda, Mariella Gastanaduy, J. Lindhe Guarisco

**Affiliations:** ^1^ Department of Otolaryngology – Head & Neck Surgery, Tulane University Medical Center, New Orleans, LA; ^2^ Department of Otolaryngology – Head & Neck Surgery, Ochsner Clinic Foundation, New Orleans, LA; ^3^ The University of Queensland Faculty of Medicine, Ochsner Clinical School, New Orleans, LA

**Keywords:** *Computed tomography*, *delayed diagnosis*, *management*, *peritonsillar abscess*, *treatment*

## Abstract

**Background:** Peritonsillar abscess (PTA) is a common occurrence in adult patients, and an important question in such often-seen disease processes is whether we are treating these patients effectively, efficiently, and economically. We sought to determine if a diagnostic computed tomography (CT) scan was associated with a difference in clinical intervention in adult patients with PTA and if CT was associated with delaying this intervention.

**Methods:** We conducted a retrospective case-control study examining therapeutic interventions in adults with PTA. Patients were divided into a control group (those diagnosed without CT, n=159) and a case group (those diagnosed with CT, n=203). Patients were examined for 3 outcomes: admission, bedside procedure (needle aspiration, incision/drainage), and surgical procedure (incision/drainage, tonsillectomy). In addition, we calculated times to admission, otolaryngology consultation, bedside procedure, and surgical procedure.

**Results:** We found a significant association between CT and intervention, with the CT group more likely to be admitted (*P*< 0.001), the non-CT group more likely to undergo a bedside procedure (*P*<0.001), and the CT group more likely to undergo operative intervention (*P*=0.02). Mean times to otolaryngology consultation, admission, and bedside procedure were significantly longer in the CT group than in the non-CT group, determined by calculating the difference of the means with 95% confidence intervals for each comparison (*P*<0.001).

**Conclusion:** We found that CT scans appear to be useful in the workup and treatment of adult patients with PTA, evidenced by significant differences in interventions between groups with and without CT scans. We also found that CT scans have the potential to delay these interventions, as the time to each intervention examined was significantly longer in patients who had a CT scan. Given the need to reduce cost, enhance efficiency, and eliminate harmful side effects (in this case, radiation exposure and delays in care), we question whether CT is the gold standard imaging method for diagnostic work up of PTA.

## INTRODUCTION

Peritonsillar abscess (PTA) is a common occurrence in adult patients, encountered not only by otolaryngologists but also by emergency medicine and primary care physicians. In the United States, PTA primarily affects patients aged 20 to 40 years, and in the latest epidemiologic data (dating back to 1995) was estimated to occur in 1 in 6,500 people each year.^1,2^ Economic burden analysis is not readily available in the literature, but given the prevalence of PTA, one can assume its significance.

An important question in such often-seen disease processes is whether we are treating these patients effectively, efficiently, and economically. Improvement in these areas benefits the healthcare system, but more importantly protects our patients from undue radiation exposure, unnecessary delays, and inappropriate medical charges. Little has been written about the impact of computed tomography (CT) scans on the diagnosis and treatment of PTA.^3,4^ Ultrasound has been increasingly used as an alternative diagnostic tool in both children and adults,^4-8^ likely reflecting clinicians’ increased attention to reducing costs and limiting radiation exposure. Comparative studies of ultrasound and CT are evidence that the imaging modality of choice is constantly being reevaluated.^8^

We sought to assess whether CT scans are associated with a difference in clinical intervention by comparing patients with PTA who underwent CT scans to patients who did not have CT scans. We assessed the potential delay in care by comparing the times to various interventions between the 2 groups.

## METHODS

The study design was approved by the Ochsner Medical Center Institutional Review Board, and waiver of patient consent was granted. Patients with a diagnosis of PTA were identified by data mining the electronic medical record and were divided into 2 groups based on the presence (CT group) or absence (non-CT group) of a diagnostic CT scan. Exclusion criteria included age <18 years, absence of true PTA (eg, patients with tonsillitis or deep neck space infection), or insufficient records to accurately establish the timing of events (ie, some archived paper records had been incorporated into the electronic medical record at a later date). The records were obtained from a single institution with several satellite campuses. Standard age and sex analysis was performed to elucidate any significant differences between the 2 groups.

Patients initially presented either to the emergency department or to their primary care physician's office from January 1, 2005 to January 1, 2016. We surveyed their medical records for 2 categorical outcomes: admission and intervention. Interventions were classified into 4 categories: bedside procedure (needle aspiration or incision and drainage), surgical procedure in the operating room (incision and drainage or quinsy tonsillectomy), no procedure, or both bedside and surgical procedures. To eliminate confounding variables, surgical procedure was defined as a surgical intervention during the same admission in which PTA was diagnosed (ie, quinsy tonsillectomy) and did not include delayed tonsillectomy or tonsillectomy planned for a later date.

Continuous variables included time to otolaryngology consultation and time to admission, bedside procedure, and surgical procedure. For continuous variables, we calculated means (shown in the tables), medians (shown in the Figure), and upper and lower quartiles. Because the continuous variables and CT scan groups were not normally distributed, Wilcoxon rank sum tests were used to test the relationship between them. We also calculated the absolute difference of the means with a 95% confidence interval (CI) for each continuous variable (eg, difference of the average time to otolaryngology consultation) in both the CT and non-CT groups. For categorical variables, we calculated frequencies and percentages for the CT and non-CT groups. Fisher exact test was used to compare proportions, with *P*<0.05 achieving significance.

**Figure.**
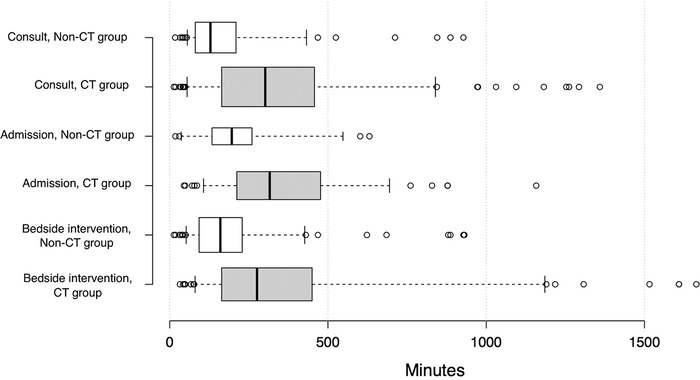
**Ungrouped box plot demonstrates the difference in time to intervention between patients who underwent computed tomography (CT group) and patients who did not (non-CT group). The center lines show the medians; box limits indicate the 25th and 75th percentiles; whiskers extend to the 5th and 95th percentiles; outliers are represented by dots; and box width is proportional to the square root of the sample size (n=133, 148, 95, 35, 191, 116 sample points, respectively).**

## RESULTS

Of the 362 total patients, 203 patients underwent CT scan as part of their initial diagnostic workup, and 159 patients were diagnosed without the aid of a CT scan. We found significant associations between CT scan and intervention (Table 1).

**Table 1.** Association of Computed Tomography (CT) Scan With Intervention (n=362)

	Non-CT Group	CT Group	
Intervention	n=159	n=203	*P* Value
Admission	35 (22.0)	101 (49.8)	<0.001
Bedside procedure	148 (93.1)	131 (64.5)	<0.001
Surgical procedure	0	10 (4.9)	0.02
Both bedside and surgical procedure	1 (0.6)	6 (3.0)	0.11
Neither bedside nor surgical procedure	10 (6.3)	56 (27.6)	<0.001

Note: Results are presented as n (%).

CT scan was positively associated with admission (*P*<0.001) and surgical procedure (*P*=0.02). Patients were more likely to be admitted and more likely to have had a surgical procedure if they underwent CT scan.

Lack of CT scan was positively associated with bedside procedure (*P*<0.001). Patients were more likely to have had a bedside procedure if they had not undergone CT scan.

CT scan was also positively associated with having neither intervention *(P<*0.001). A subset of patients did not undergo any procedural intervention; this situation was more likely to occur in patients who had a CT scan.

We found no significant association between the 2 groups in the number of patients who underwent both bedside and surgical procedures (*P*=0.11), but the n in this subgroup is small with only 1 patient in the non-CT group and 6 patients in the CT group.

The times to all interventions, with the exception of surgical procedure, was significantly longer in the CT group (Table 2). Mean time to otolaryngology consultation in the non-CT group was 177 minutes vs 352 minutes in the CT group for an absolute difference of 175 minutes (95% CI, 129-221, *P*<0.001). Mean time to admission in the non-CT group was 223 minutes vs 359 minutes in the CT group for an absolute difference of 136 minutes (95% CI, 99.3-173, *P*<0.001). Mean time to bedside procedure in the non-CT group was 195 minutes vs 377 minutes in the CT group for an absolute difference of 182 minutes (95% CI, 126-238, *P*<0.001).

**Table 2.** Time to Intervention by Treatment Group

	Non-CT Group	CT Group		
Intervention	n=159	n=203	Difference of the Means	*P* Value
Mean time to otolaryngology consultation, minutes	177 [80.5-210]	352 [164-460]	175 (95% CI, 129-221)	<0.001
Mean time to admission, minutes	223 [132-262]	359 [211-478]	136 (95% CI, 99.3-173)	<0.001
Mean time to bedside procedure, minutes	195 [92.5-232]	377 [164-450]	182 (95% CI, 126-238)	<0.001
Mean time to surgical procedure, days	0.21^a^	1.28 [0.48-2.03]	1.07 (95% CI, 0.902-1.24)	0.13

^a^Calculated using nonparametric test due to the presence of only one data point.

Note: Data are presented as mean [lower quartile-upper quartile] unless otherwise indicated.

CI, confidence interval; CT, computed tomography.

Time to surgical procedure was 0.21 day in the non-CT group vs 1.28 days in the CT group, yielding an absolute difference of 1.07 days (95% CI, 0.902-1.24, *P*=0.13). A nonparametric test was used for the surgical procedure analysis because the non-CT group had one data point (only one patient underwent surgical procedure in the non-CT group). With one data point in this subgroup, the difference is not a true difference of the means and was therefore excluded from the box plot showing time to intervention (Figure).

We found no difference in sex between the 2 groups with a 57.8% male cohort in the non-CT group and a 55.7% male cohort in the CT group (*P*=0.68). Mean ages in the non-CT and CT groups were 31.3 years (range, 18-76 years) and 34.9 years (range, 18-83 years), respectively, for an absolute difference of 3.6 years (95% CI, 0.62-6.6, *P*<0.001), indicating that older age was associated with CT scan (Table 3).

**Table 3.** Age and Sex Distribution by Treatment Group

	Non-CT Group	CT Group		
Intervention	n=159	n=203	Difference of the Means	*P* Value
Mean age, years (range)	31.3 (18-76)	34.9 (18-83)	3.6 (95% CI, 0.62-6.6)	<0.001
Male, n (%)	92 (57.8)	113 (55.7)	N/A	0.68

CI, confidence interval; CT, computed tomography; N/A, not applicable.

## DISCUSSION

CT scan is commonly used as part of the initial workup of patients with potential PTA. Studies have sought to elucidate physical examination findings and characteristic imaging findings to aid in diagnosis.^9,10^ Trismus, uvular deviation, and palatal edema,^9^ as well as tonsillar asymmetry and muffled voice,^10^ are among the most recent reports of clinical findings that are demonstrably statistically significant in their association with PTA, albeit in studies with relatively low statistical power. Considering abscess characteristics on CT scans, 2 Japanese studies demonstrated that PTA is most likely to be found in the intracapsular space and along the superior aspect of the tonsillar pole.^11,12^ While obtaining the maximum benefit from physical examination and CT scan as diagnostic tools is desirable, the question has been raised whether the best imaging modality is something different.^9-11,13^

As early as 1994, researchers explored ultrasound as a potential diagnostic tool for adult PTA.^14-17^ However, as radiologic technology improved, a trend for increasing CT utilization was established, with some studies from the same mid-1990s time period favoring CT as a relatively new and evolving technique.^18-20^ Currently, at our institution, CT is still used almost exclusively for PTA workup. Meanwhile, academic discussion surrounding utilization of ultrasound in PTA has continued in earnest, with a 2017 expert opinion article emphasizing that ultrasound may be a favorable alternative to CT.^7^

Many of these discussions have focused on children, a population in whom minimizing radiation exposure is important.^5,6^ Huang et al found that transcervical ultrasound was safe and effective in the diagnosis of pediatric PTA.^5^ Their examination of 179 patients revealed no significant difference in readmission rates between children diagnosed with transcervical ultrasound vs the standard method of diagnosis, a combination of physical examination and CT scan. They did, however, find a statistically significant decrease in radiation exposure between the 2 groups.

Diagnostic accuracy may not be sacrificed by using ultrasound rather than CT. Initially, ultrasound was thought to have poorer sensitivity for PTA, but recent randomized controlled trials and cohort studies show that this may not be the case. In a 2012 randomized controlled trial of 28 patients, Costantino et al showed better diagnostic accuracy, a higher rate of successful needle aspirations, shorter length of hospital stay, and fewer otolaryngology consultations for patients who underwent ultrasound as a part of their diagnostic workup.^8^ One year later, Biron et al demonstrated the cost benefit of ultrasound-guided drainage vs incision and drainage in the operating room and a shorter length of hospital stay.^21^ Several cohort studies also reinforce these findings, in both pediatric and adult patients, and using both transcervical and intraoral ultrasound methods.^22-24^ Ever-evolving ultrasound technology and technique likely contribute to the favorable outcomes with ultrasound-guided management.^25,26^

While ultrasound use is increasing, ambiguity concerning the best diagnostic tool for PTA remains.^4^ Regional differences undoubtedly exist, and personal preferences vary by physician. Our study did not address ultrasound as a diagnostic tool simply because it is not done at our institution, but the recent literature reinforces that a wide variety of management strategies exist. While we did not have the opportunity to objectively assess ultrasound vs CT vs no imaging in this study, our findings contribute to the overall movement of finding the best diagnostic algorithm for patients with PTA. In this study, clinicians presumably found CT to be useful because it was significantly associated with different interventions. For example, patients who underwent CT were more likely to be admitted, less likely to undergo bedside incision and drainage, and more likely to undergo surgical intervention. These results can be interpreted as CT scans having an effect on the chosen clinical intervention. However, every intervention, with the exception of surgery, was significantly delayed in the CT group, including time to otolaryngology consultation. While CT scans may be effective in diagnosing adult peritonsillar infections, questions remain: Are CT scans the most cost-effective, time-efficient, and safest route of diagnosis? Can ultrasound potentially replace CT scan in the appropriately selected patient?

An important consideration in deep neck space infections is that PTA may mimic or be difficult to discern from an infection in an adjacent deep neck space. CT scan in this situation would be ideal to assess the parapharyngeal, retropharyngeal, and prevertebral spaces, which can be associated with more severe complications such as airway compromise, Lemierre syndrome, jugular vein thrombosis, and mediastinitis. Such a patient may present with a more toxic appearance than a patient with an isolated PTA, including fever, trismus, decreased range of motion of the neck, and tender cervical lymphadenopathy.^27-30^

Limitations of this study are those inherent to a retrospective design. Although generalizations can be drawn from the data, a closer investigation into each individual case would elucidate how an individual's presentation may have guided imaging decisions and how imaging may have in turn guided treatment decisions. For instance, the cases in which imaging was used were possibly more complex than the cases of the patients who did not undergo imaging, and because of that complexity, a greater amount of time was dedicated to those patients’ care, giving an appearance of intervention delay. Nevertheless, we believe this study is valuable in supporting the role of imaging in the diagnosis of adult PTA and that further investigation to identify the ideal imaging modality would be extremely valuable.

## CONCLUSION

We found that CT scan appears to be useful in the workup and treatment of adult PTA, evidenced by significant differences in interventions between groups with and without CT scans. We also found that CT scans have the potential to delay these interventions, as time to each intervention was significantly longer in patients who underwent CT. Given the need to reduce cost, enhance efficiency, and eliminate harmful side effects (in this case, radiation exposure and delays in care), we question whether this imaging method is truly the gold standard for diagnostic workup of PTA. Increasingly, clinicians are documenting the utility of ultrasound as an alternative to CT, but we should also not forget the value of a thorough history and physical examination. Keeping in mind that CT has the potential to cause delays in care, and knowing that its costs are not insubstantial, a reasonable next step may be comparing patients with PTA undergoing CT vs ultrasound vs no imaging, assessing the interventions chosen for their management, and examining the time to each of those interventions, with a cost analysis between the groups.

## Notes

This article meets the Accreditation Council for Graduate Medical Education and the American Board of Medical Specialties Maintenance of Certification competencies for Patient Care and Medical Knowledge.
